# How to Deprive Iodine of Its Stain

**Published:** 1877-11

**Authors:** 


### HOW TO DEPRIVE IODINE OF ITS STAIN.

Add a few drops of carbolic acid to the tincture and it will not stain; moreover the tincture is more efficacious and its action more certain. M. Boggs recommends the following formula for use in injections: Alcholic tincture of iodine, 3 grammes; carbolic acid, 6 drops; glycerine, 30 grammes; distilled water, 150 grammes.—*Ex. Am. Jl. Med. Sciences.*

